# Transmission and Maintenance Cycle of *Bartonella quintana* among Rhesus Macaques, China

**DOI:** 10.3201/eid1902.120816

**Published:** 2013-02

**Authors:** Hao Li, Wei Liu, Guang-Zhou Zhang, Zhao-Zeng Sun, Jie-Ying Bai, Bao-Gui Jiang, Yao-Yun Zhang, Xiao-Guang Zhao, Hong Yang, Guang Tian, Yu-Chuan Li, Lin Zeng, Michael Kosoy, Wu-Chun Cao

**Affiliations:** Author affiliations: State Key Laboratory of Pathogen and Biosecurity—Beijing Institute of Microbiology and Epidemiology, Beijing, People’s Republic of China (H. Li, W. Liu, B.-G. Jiang, Y.-Y. Zhang, X.-G. Zhao, H. Yang, G. Tian, Y.-C. Li, W.-C. Cao);; Academy of Military Medical Sciences Laboratory Animal Center, Beijing (G.-Z. Zhang, Z.-Z. Sun, J.-Y. Bai, L. Zeng);; Centers for Disease Control and Prevention, Fort Collins, Colorado, USA (M. Kosoy)

**Keywords:** Bartonella quintana, rhesus macaques, reservoir host, lice, transmission, China, vector-borne infections, Bartonella

## Abstract

We detected *Bartonella quintana* in 48.6% of captive rhesus macaques from an animal facility in Beijing, China. Prevalence of infection increased over the period of observation. Our findings suggest that macaques may serve as reservoir hosts for *B. quintana* and that *Pedicinus obtusus* lice might act as efficient vectors.

*Bartonella quintana* is a vector-transmitted, hemotropic, and extremely fastidious gram-negative bacterium. Infection with *B. quintana* has been recognized to cause a broad spectrum of disease, including trench fever, chronic bacteremia, endocarditis, and bacillary angiomatosis (*1*–*4*). Humans are the primary reservoir host for *B. quintana*, which, unlike most other *Bartonella* species, lacks an identified animal reservoir, although some recent reports have found *B. quintana* in dogs and in cynomolgus and rhesus macaques (*5*–*7*). Almost 60 years ago, rhesus macaques were able to be experimentally infected with *B. quintana* (*8*). However, nonhuman primates have not been shown to support long-term maintenance, multiplication, and transmission of this pathogen, all of which would be expected if these animals were to act as reservoir species. Observations of monkey ectoparasites transmitting *B. quintana* between nonhuman primates or infecting humans have also not been reported.

## The Study

A laboratory animal surveillance program intended to screen for the presence of adventitious pathogens was performed at the Laboratory Animal Center of the Academy of Military Medical Sciences, Beijing, China. Four blood samples from 10 captive-bred rhesus macaques (*Macaca mulatta*) were presumed to be infected with *Bartonella* spp. according to Giemsa-stained smears and transmission electron microscopy (Technical Appendix Figure 1). Further PCR and sequence analysis of 3 gene targets (internal transcribed spacer [ITS], *gltA*, and *rnp*B) confirmed the existence of *B. quintana* in the 4 parasite-positive macaques (Technical Appendix Table 1). In addition, *B. quintana* was successfully isolated from the 4 monkeys by blood plating.

**Figure 1:**
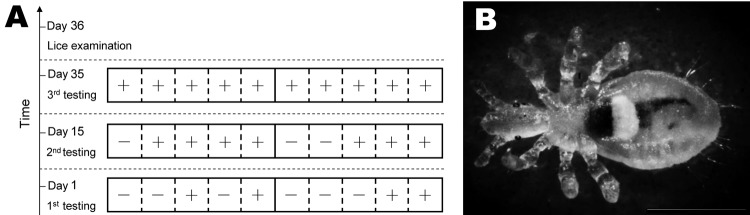
Monitoring surveillance of *Bartonella quintana* infection in macaques and identification of lice. A) Macaques were housed in linked cages (squares); dashed lines indicate wired net enabling direct contact between macaques, and solid line indicates wall that does not enable contact between macaques. +, positive result for PCR; –, negative result for PCR. B) Stereomicroscope image of a *Pedicinus obtusus* louse, a macaque-specific ectoparasite characterized by a slender body ≈1.0–3.0 × 0.5–1.0 mm; long, sharp claws in distal end of 6 legs of the same length; and a plurality of rows of small hairs on both sides of the abdomen. Scale bar indicates 500 μm.

During the 36-day period of observation, 3 screening tests of the 10 macaques showed an increasing prevalence of *B. quintana*: 4 were found positive at day 1, 7 positive at day 15, and all 10 positive at day 35 (Figure 1, panel A). Close examination of the monkeys revealed no skin scratch or wound indicative of direct contact between them.

**Figure 2:**
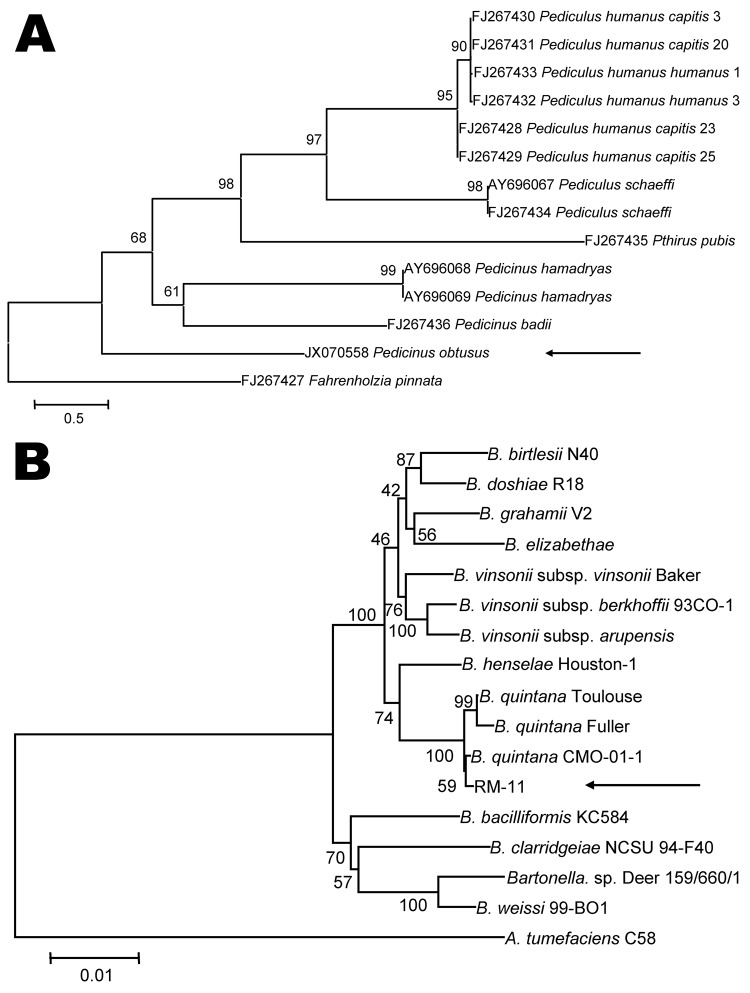
Phylogenetic analyses of louse species and *Bartonella* spp. A) Phylogenetic tree of louse species based on the partial Cytb sequence (364-bp), obtained by using the neighbor-joining method with maximum composite likelihood analysis and bootstrap analysis of 1,000 replicates. Arrow indicates the *Pedicinus obtusus* louse identified in this study. The tree was rooted with the louse species *Fahrenholzia pinnata*. Numbers shown at each node indicate percentage of replicates that reproduced the topology of each clade. Scale bar indicates estimated evolutionary distance of 0.5 substitutions per position. B) Phylogenetic tree of *Bartonella* spp. based on the combined RNase P RNA, 16S, and 23S rRNA sequence alignment (4131-bp), obtained by using the same analytical method as described in panel A. Arrow indicates the RM-11 isolate. The tree was rooted with the louse species *Agrobacterium tumefaciens*. The GenBank accession numbers of *Bartonella* strains used for phylogenetic analysis are shown in Technical Appendix Table 2. Scale bar indicates estimated evolutionary distance of 0.01 substitutions per position.

Examination for ectoparasites at the last day of observation (day 36) revealed that all 10 monkeys were infested with lice (mean 10.3 lice/monkey, range 4–28 lice). Lice from each infested monkey were combined in 2 pools. *B. quintana* was identified in all pools of lice by PCR selective for ITS, *glt*A, and *rnp*B. Partial Cytb sequence (660-bp) of the louse was obtained (GenBank accession no. JX070558) (Technical Appendix Table 1); phylogenetic analysis of Cytb identified the louse as a relative of lice of the genus *Pedicinus* (Figure 2, panel A). By means of stereomicroscopy, the louse was then identified as *Pedicinus obtusus* (Figure 1, panel B), a macaque-specific ectoparasite, according to morphologic criteria (*9*,*10*).

Of the 60 rhesus macaques (27 male, 33 female) housed in 5 other rooms in individual cages in the same facility, an additional 30 were found to be positive for *B. quintana* by PCR. *B. quintana* prevalence among sexually immature macaques was higher than that among sexually mature macaques, but this difference was not significant (29/54 [53.7%] vs. 1/6 [16.7%], respectively; p = 0.195). *B. quintana* prevalence among male macaques was similar to that among females (15/27 [55.6%] vs. 15/33 [45.5%], respectively).

Nucleotide sequences of ITS (123-bp), *gltA* (539-bp), and *rnpB* (336-bp) from all macaques and pools of lice were identical; they differed from from those of *B. quintana* strain Toulouse by1–3 bp . Phylogenetic markers *rnpB*, 16S rRNA, and 23S rRNA (*11*) were amplified and sequenced from the strain identified in this study (RM-11) (Technical Appendix Table 1). Phylogenetic analysis of their combined sequence alignment placed the RM-11 strain on a separate branch along with the strain of *B. quintana* from a cynomolgus macaque and in the same clade as strains from patients in Europe who had trench fever (strains Toulouse and Fuller) (Figure 2, panel B).

To evaluate the ability of the isolate to cause disease, we intravenously inoculated 4 *Bartonella* spp.–negative rhesus macaques with *B. quintana* isolated from a blood sample of a macaque from this study and twice passaged on agar (detailed methods described in the Technical Appendix). Bacteremia reached a peak in 1 monkey on day 7 postinoculation (160 CFU/mL), in 2 monkeys on day 14 postinoculation (290 and 240 CFU/mL), and in 1 monkey on day 42 postinoculation (240 CFU/mL). Bacteremia then dropped to below a detectable level after 15 weeks postinoculation for all monkeys (Technical Appendix Figure 2). A relapsing pattern of bacteremia was observed during the experiment. Rectal temperature, hemogram, and blood biochemistry results for the 4 monkeys remained within normal limits.

The animal facility employees who had direct contact with monkeys during cage cleaning and feeding activities were tested for *B. quintana* infection. Paired serum samples collected at 2 time points 3 months apart were tested for IgG against *B. quintana* by indirect immunofluorescence assay, as described (*12*). The baseline serum samples were all negative at a dilution of 1:64. Among the serum samples collected 3 months later, 3 had IgG titers of 256, 1 had a titer of 512, and 4 were negative. For all blood samples collected at the 2 time points, PCR detection, blood-smear staining, and blood culture for *Bartonella* spp. were negative. Analysis of questionnaires revealed that all 4 of the workers with evidence of seroconversion reported lice exposure; 2 of them were scratched or bitten by monkeys.

## Conclusions

We demonstrated high prevalence of *B. quintana* in a colony of rhesus macaques and postulated the transmission among macaques by *P. obtusus* lice. Our findings suggest that macaques are susceptible to *B. quintana* infection and can sustain vector infection and subsequent transmission. In addition, rhesus macaques showed long-lasting chronic bacteremia without apparent clinical abnormalities after experimental inoculation, suggesting a high level of adaptation of the pathogen to macaques.

It is unknown how the macaques were initially exposed to infected lice or how the lice became infected with *B. quintana.* We postulate that the lice became infected with *B. quintana* from an infected macaque and thereafter acted as efficient vectors among the rest of the macaques in the colony. We cannot completely exclude the possibility that transmission occurred through direct contact between macaques within the colony; however, we did not observe any skin scratches or wounds on the animals.

Four workers involved with care of the macaques showed seroconversion to antigens derived from our strain. This finding may relate to exposure to the *P. obtusus* lice found on macaques in the animal facility; however, we cannot exclude the possibility of direct contact with these animals as the mode of *Bartonella* spp. transmission because information obtained from the questionnaires indicates that 2 of the 4 seropositive workers were scratched or bitten by the colony’s macaques. No clinical signs were observed from these persons, indicating an asymptomatic course of *B. quintana* infection, which has been frequently reported in other studies (*1*,*13*). However, we cannot exclude the possibility that bacteremia continued for an extended time in humans, because the animal care personnel were sampled only 2 times, so we might have missed bacteremia of short duration. Bacterial levels obtained after monkey inoculation was low compared with levels in the initial inoculum, a finding similar to that of a previous study (*14*).

In summary, our findings suggest that the macaques might serve as reservoir hosts for *B. quintana* and that lice might act as efficient vectors. Our data also indicate that macaques could be a source for human infection with *B. quintana*. Further research is needed to understand the underlying mechanism of *B. quintana* transmission by the *P. obtusus* louse.

Technical Appendix Detailed methods of inoculation of 4 *Bartonella* spp.–negative rhesus macaques with isolate from captive rhesus macaques in Beijing; nucleotide sequence of primers used for PCR analysis; GenBank accession numbers of *Bartonella* strains used for phylogenetic analysis; analysis of thin-film blood smear and peripheral blood from rhesus macaque; and timeline of bloodstream infections of *B. quintana* strain RM-11 in 4 rhesus macaques.
