# Supplementary material for: Transmission and Maintenance Cycle of Bartonella quintana among Rhesus Macaques, China
Source: Emerg Infect Dis. 2013 Feb;19(2):297–300. doi: 10.3201/eid1902.120816 (PMC3563275; doi:10.3201/eid1902.120816)
Supplement: Technical Appendix — Detailed methods of inoculation of 4 Bartonella spp.–negative rhesus macaques with isolate from captive rhesus macaques in Beijing; nucleotide sequence of primers used for PCR analysis; GenBank accession numbers of Bartonella strains used for phylogenetic analysis; analysis of thin-film blood smear and peripheral blood from rhesus macaque; and timeline of bloodstream infections of B. quintana strain RM-11 in 4 rhesus macaques. [file 12-0816-Techapp-s1.pdf]

Article DOI: http://dx.doi.org/10.3201/eid1902.120816

Transmission and Maintenance Cycle of *Bartonella quintana* among Rhesus Macaques, China

Technical Appendix

Bartonella DNA detection

PCR analyses were performed at the Beijing Institute of Microbiology and Epidemiology. Briefly, DNA was extracted from blood specimens using QIAmp DNA Mini Kit (Qiagen). PCR targeting the 16S-23S internal transcribed spacer region (ITS), citrate synthase (*gltA*), and RNase P RNA (*rnpB*) genes were carried out (Technical Appendix Table 1), with *B. grahamii* as a positive control and sterile deionized water as a negative control. A positive result was determined only when all three targets were amplified. For positive samples, 16S rDNA-encoding and 23S rDNA-encoding genes were additionally amplified for further phylogenetic characterization (Technical Appendix Table 1). Short amplified DNA fragments (<800-bp) were directly sequenced in both directions, and long DNA fragments were cloned into pGEM-T easy vector system (Promega) for sequencing on an automated DNA sequencer (3730 DNA Sequencer, Applied Biosystems). To reduce contamination, DNA extraction reagent setup and amplification were performed in separate rooms. Certified DNA/RNase-free filter barrier tips were used to prevent aerosol contamination.

Cytb gene amplification

Pooled (2–14 lice/pool) lice samples were homogenized in sterile phosphate buffered saline with a Bullet Blender (NextAdvance Inc., Averill Park, NY, USA). DNA was extracted

from the homogenates using DNeasy Tissue Kit (Qiagen) according to manufacturer's instructions. For phylogenetic characterization of the louse, a portion of cytochrome b gene (Cytb) was amplified and sequenced with primers Cytb-f and Cytb-r (Appendix Table 1).

Inoculation of naive rhesus macaques

Four rhesus macaques confirmed to be *Bartonella*-negative by morphologic examination, blood test by nested PCR and serum test by indirect immunofluorescence assay were selected (all tests repeated after a week's interval), deloused, and held in a clean room for 7 days before the inoculation. The macaques were intravenously inoculated with isolate of *B. quintana* that was originally isolated from a blood sample of a macaque from this study and twice passaged on agar. Peripheral blood was collected post inoculation weekly in EDTA vacuum tubes. The frozen-thawed blood specimens were plated in duplicates on chocolate agar. Colony forming units (CFUs) were counted on day 15 after plating. During the whole observation period, rectal morning temperature was taken daily; laboratory routine tests of hemogram (leukocyte, erythrocyte, lymphocyte, granulocytes, platelet, hemoglobin and hematocrit) and blood biochemistry (alkaline phosphatase, alanine transaminase, aspartate aminotransferase, lactate dehydrogenase, albumin, urea nitrogen, cholesterol, triglyeride, glucose, creatine kinase, creatinine) were performed every third day.

IFA serology

Colonies of *B. quintana* harvested after 5 days of the growth on a chocolate agar plate were inoculated onto Vero E6 cells. After 3 days, the infected cells were harvested for antigen preparation. Slides of culture cells were fixed in a 1:1 solution of acetone and methanol and prepared for indirect immunofluorescence assay (IFA). Briefly, all serum samples were diluted 1:64 in PBS, overlaid onto antigen-containing slides, incubated at 37°C for 30 minutes, washed, and incubated at 37°C for 30 minutes with goat antihuman immunoglobulin G conjugated with

fluorescein isothiocyanate. Positive samples were then tested with additional serial dilutions. A serum with antibodies against *B. quintana* (Euroimmun, Lubeck, Germany) was used as a positive control for IFA.

Reference

1. Jensen WA, Fall MZ, Rooney J, Kordick DL, Breitschwerdt EB. Rapid identification and differentiation of *Bartonella* species using a single-step PCR assay. J Clin Microbiol. 2000;38:1717–22. PubMed

2. Brouqui P, Lascola B, Roux V, Raoult D. Chronic *Bartonella quintana* bacteremia in homeless patients. N Engl J Med. 1999;340:184–9. PubMed http://dx.doi.org/10.1056/NEJM199901213400303

3. Birtles RJ, Raoult D. Comparison of partial citrate synthase gene (gltA) sequences for phylogenetic analysis of *Bartonella* species. Int J Syst Bacteriol. 1996;46:891–7. PubMed http://dx.doi.org/10.1099/00207713-46-4-891

Technical Appendix Table 1. Nucleotide sequence of primers used for PCR analysis

Target	Primer	Primer sequence (5′ → 3′)	Reference
ITS	302F	YCTTCGTTTCTCTTTCTTCA	(*1*)
	473R	AACCAACTGAGCTACAAGCC	
	311F	CTCTTTCTTCAGATGATGATCC	
	448R	GGATAAACCGGAAAACCTTC	
gltA	CS140f	TTACTTATGATCCKGGYTTTA	(*2*,*3*)
	CS 443	GCTATGTCTGCATTCTATCA	
	CS 979	TGCATGATTTTTGCACGTGG	
rnpB	rnpB-Fo	AGTCGGCTGGGCAACCGCGC	This study
	rnpB-Ro	GCCTGTAAGCCGGGTTCTGTA	
	rnpB-Fi	GCAAGTGAGGAAAGTCCG	
	rnpB-Ri	TGTAAGCCGGGTTCTGTA	
16S rDNA	16S-F1	ACTGTCTCATAATGAGGTAGAGGC	This study
	16S-R1	AGATTTCGGAAAGAATATGGCG	
	16S-F2	GATTTAGCGTCATATGCATGGTT	
	16S-R2	ATATGTTCTCGTCGATTCAAGC	
23S rDNA	23S-Fo	tttgtgagtgatgctctatgcg	This study
	23S-Ro	AGAAGCTGGTCTTTTCTGCTG	
	23S-Fi	ccataaccaccaagtcagcaa	
	23S-Ri	TCCTGGAGGTATCGGAAGTGA	
	23S-Fii	aagaccttacaatacacgcaatc	
	23S-Rii	CAAAGAATGCCGACAAACATTG	
Cytb	Cytb-f	GCTACTCATTATGARKCTTC	This study
	Cytb-r	TCTGGYTGRATATGAGGWGGWGT	

Technical Appendix Table 2. GenBank accession numbers of *Bartonella* strains used for phylogenetic analysis in this study*

Species/strain	*rnpB*	16S rDNA	23S rDNA
B. bacilliformis KC584	AF440224	AF442955	L39095
B. birtlesii N40	AF441292	AF204274	AF410944
B. clarridgeiae NCSU 94-F40	AY033649	U64691	AF410938
B. doshiae R18	AF441294	Z31351	AF410939
B. elizabethae F9251	AY033770	L01260	AF410940
B. grahamii V2	AF441293	Z31349	AF410942
B. henselae Houston-1	AY033897	M73229	AF410943
B. quintana CMO-01–1	AY484594	AY484592	AY484593
B. quintana Fuller	AY033948	M11927	AF410946
B. quintana Toulouse	BX897700	BX897700	BX897700
B. Vinsonii subsp. *arupensis*	AF441295	AF214558	AF410937
B. vinsonii subsp. *vinsonii* Baker	AY033502	Z31352	AF411589
B. vinsonii subsp. *berkhoffii* 93CO-1	AF375873	L35052	AF410941
B. weissi 99-BO1	AF376050	AF291746	AF410947
Bartonella. sp. Deer 159/660/1	AF376051	AF373845	AF410945
RM-11	JQ314421*	JQ314414*	JQ314415*

*GenBank accession numbers for sequences of the *rnpB*, 16S rDNA, and 23S rDNA are listed. Sequences data of the strain isolated in the study were deposited in GenBank under the accession numbers indicated by asterisks.

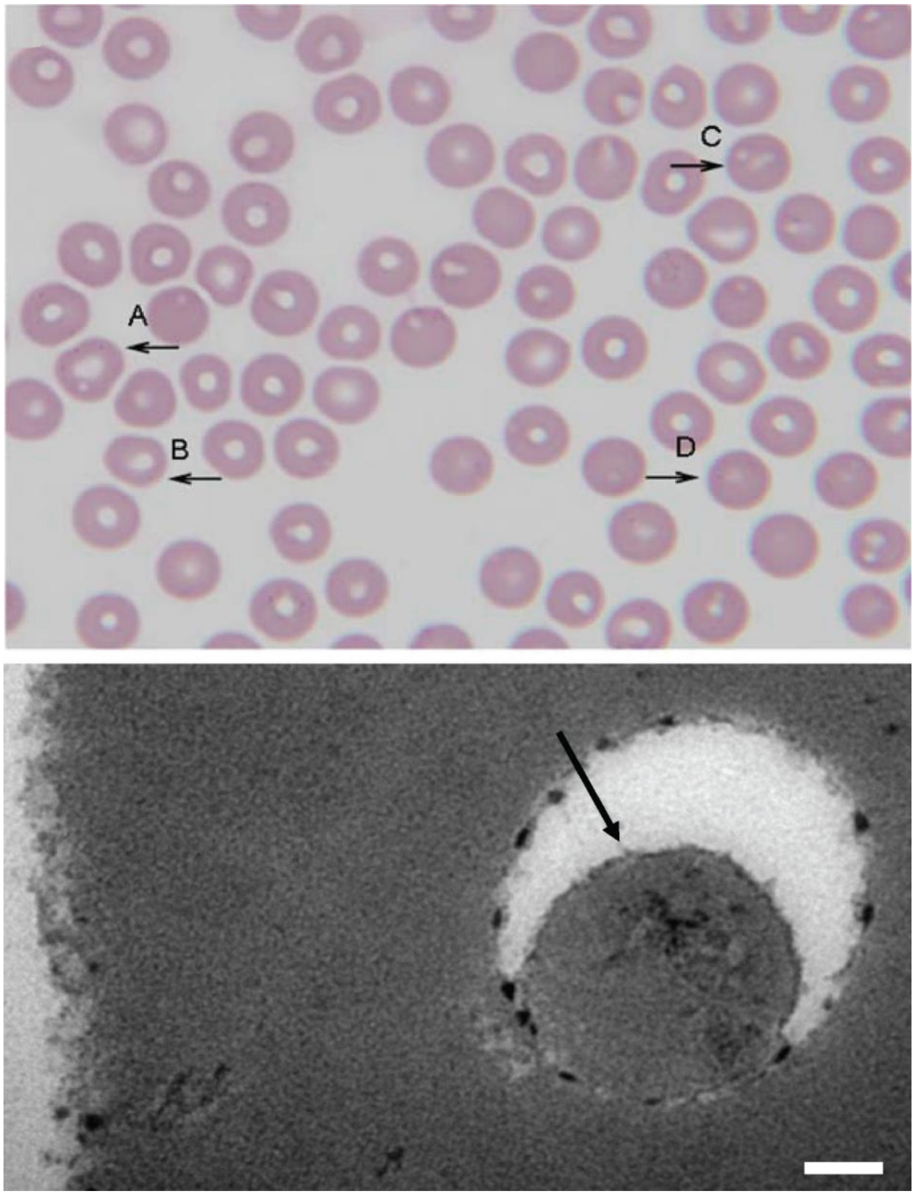

Technical Appendix Figure 1. Top: Giemsa stain of thin-film blood smear. A) Suspected intraerythrocytic corpuscles; B) Suspected member-associated corpuscles; C) Stomatocyte; D) Spherostomatocyte with suspect intraerythrocytic corpuscles. Bottom: Observation of peripheral blood from rhesus macaque by transmission electron microscopy shows erythrocyte with vacuole-enclosed suspected organism. Scale bar indicates 100 nm.

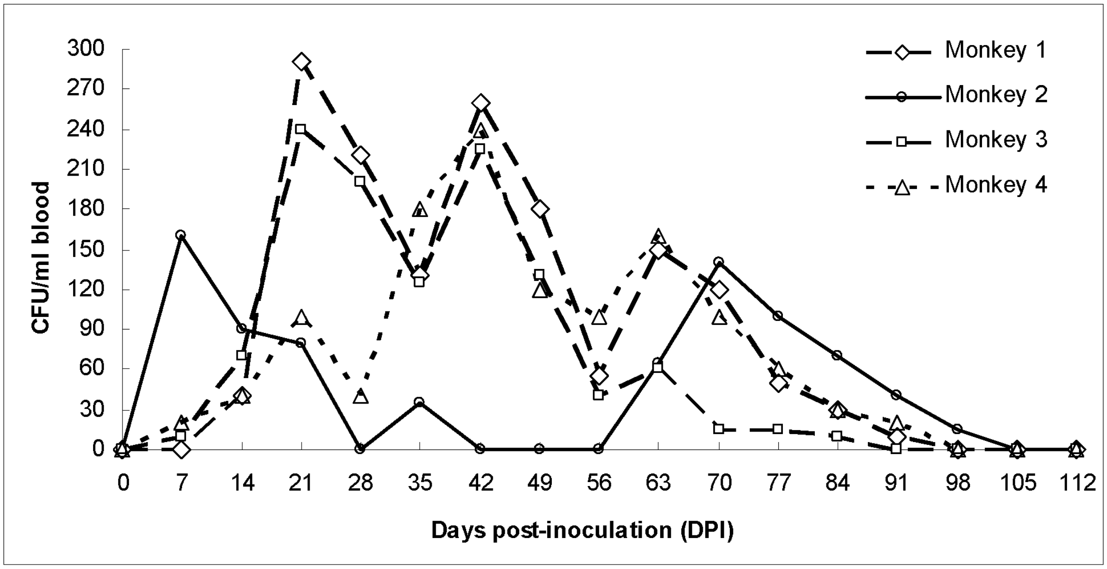

Technical Appendix Figure 2. Timeline of bloodstream infections of strain RM-11 in 4 rhesus macaques. Blood cultures were performed in duplicate weekly for 112 days. CFU of *B. quintana* bacteria per ml of blood was counted on day 15 after blood plating.
